# Supplementary material for: Transcriptome Analysis Reveals Vimentin-Induced Disruption of Cell–Cell Associations Augments Breast Cancer Cell Migration
Source: Cells. 2022 Dec 13;11(24):4035. doi: 10.3390/cells11244035 (PMC9776984; doi:10.3390/cells11244035)
Supplement: Supplementary file 1 [file cells-11-04035-s001.zip › DEGs and enrichment analysis.zip!/DEGs and enrichment analysis/WT_VIMvsVector KEGG/GSEA/WT_VIM/MISMATCH_REPAIR/MISMATCH_REPAIR(HSA03430).html]

Details for gene set MISMATCH\_REPAIR(HSA03430)[GSEA]

|  || Dataset | fpkm.sample |
| Phenotype | sample.cls |
| Upregulated in class | WT\_VIM |
| GeneSet | MISMATCH\_REPAIR(HSA03430) |
| Enrichment Score (ES) | 0.66907376 |
| Normalized Enrichment Score (NES) | 1.1574947 |
| Nominal p-value | 0.0 |
| FDR q-value | 0.2930764 |
| FWER p-Value | 0.498 |
Table: GSEA Results Summary

  

Fig 1: Enrichment plot: MISMATCH\_REPAIR(HSA03430)      
 Profile of the Running ES Score & Positions of GeneSet Members on the Rank Ordered List

  

| PROBE | DESCRIPTION (from dataset) | GENE SYMBOL | GENE\_TITLE | RANK IN GENE LIST | RANK METRIC SCORE | RUNNING ES | CORE ENRICHMENT || 1 | ENSG00000228546 | AC091390.2 |  |  | 903 | Infinity | -0.0151 | Yes |
| 2 | ENSG00000233448 | PMS2P9 |  |  | 2086 | Infinity | -0.0349 | Yes |
| 3 | ENSG00000062822 | POLD1 |  |  | 3291 | 3.989 | 0.0186 | Yes |
| 4 | ENSG00000106628 | POLD2 |  |  | 3557 | 3.585 | 0.0805 | Yes |
| 5 | ENSG00000049541 | RFC2 |  |  | 3833 | 3.234 | 0.1358 | Yes |
| 6 | ENSG00000105486 | LIG1 |  |  | 4197 | 2.863 | 0.1827 | Yes |
| 7 | ENSG00000174371 | EXO1 |  |  | 4616 | 2.604 | 0.2238 | Yes |
| 8 | ENSG00000132646 | PCNA |  |  | 4836 | 2.473 | 0.2659 | Yes |
| 9 | ENSG00000133119 | RFC3 |  |  | 6033 | 2.025 | 0.2832 | Yes |
| 10 | ENSG00000117748 | RPA2 |  |  | 7021 | 1.813 | 0.3001 | Yes |
| 11 | ENSG00000076242 | MLH1 |  |  | 7592 | 1.696 | 0.3218 | Yes |
| 12 | ENSG00000106399 | RPA3 |  |  | 7836 | 1.651 | 0.3483 | Yes |
| 13 | ENSG00000116062 | MSH6 |  |  | 7992 | 1.625 | 0.3758 | Yes |
| 14 | ENSG00000111445 | RFC5 |  |  | 8152 | 1.601 | 0.4027 | Yes |
| 15 | ENSG00000132383 | RPA1 |  |  | 8219 | 1.591 | 0.4311 | Yes |
| 16 | ENSG00000163918 | RFC4 |  |  | 8301 | 1.578 | 0.4589 | Yes |
| 17 | ENSG00000269534 | AC011466.3 |  |  | 8453 | 1.552 | 0.4851 | Yes |
| 18 | ENSG00000276840 | PMS2P10 |  |  | 8615 | 1.533 | 0.5108 | Yes |
| 19 | ENSG00000095002 | MSH2 |  |  | 8801 | 1.507 | 0.5356 | Yes |
| 20 | ENSG00000077514 | POLD3 |  |  | 9747 | 1.389 | 0.5453 | Yes |
| 21 | ENSG00000278416 | PMS2P2 |  |  | 9866 | 1.375 | 0.5688 | Yes |
| 22 | ENSG00000204086 | RPA4 |  |  | 11238 | 1.244 | 0.5686 | Yes |
| 23 | ENSG00000078319 | PMS2P1 |  |  | 11775 | 1.204 | 0.5819 | Yes |
| 24 | ENSG00000127957 | PMS2P3 |  |  | 12746 | 1.120 | 0.5862 | Yes |
| 25 | ENSG00000273897 | AC211476.3 |  |  | 14503 | 0.996 | 0.5749 | Yes |
| 26 | ENSG00000035928 | RFC1 |  |  | 14751 | 0.986 | 0.5890 | Yes |
| 27 | ENSG00000229018 | PMS2P7 |  |  | 14774 | 0.984 | 0.6068 | Yes |
| 28 | ENSG00000273598 | AC211486.3 |  |  | 15347 | 0.950 | 0.6148 | Yes |
| 29 | ENSG00000119684 | MLH3 |  |  | 15437 | 0.943 | 0.6307 | Yes |
| 30 | ENSG00000122512 | PMS2 |  |  | 15906 | 0.926 | 0.6399 | Yes |
| 31 | ENSG00000067601 | PMS2P4 |  |  | 15974 | 0.921 | 0.6559 | Yes |
| 32 | ENSG00000106028 | SSBP1 |  |  | 16461 | 0.890 | 0.6641 | Yes |
| 33 | ENSG00000187953 | PMS2CL |  |  | 17101 | 0.850 | 0.6691 | Yes |
| 34 | ENSG00000175482 | POLD4 |  |  | 17979 | 0.800 | 0.6691 | No |
| 35 | ENSG00000113318 | MSH3 |  |  | 18997 | 0.742 | 0.6656 | No |
| 36 | ENSG00000174384 | PMS2P6 |  |  | 23678 | 0.464 | 0.5950 | No |
| 37 | ENSG00000277873 | AC131159.2 |  |  | 27133 | 0.080 | 0.5380 | No |
| 38 | ENSG00000258392 | RPA2P1 |  |  | 27356 | 0.000 | 0.5342 | No |
| 39 | ENSG00000241350 | PMS2P11 |  |  | 28249 | 0.000 | 0.5191 | No |
| 40 | ENSG00000123965 | PMS2P5 |  |  | 29010 | 0.000 | 0.5063 | No |
| 41 | ENSG00000216475 | AL024474.1 |  |  | 30460 | 0.000 | 0.4817 | No |
| 42 | ENSG00000233526 | RFC5P1 |  |  | 33745 | NaN | 0.4263 | No |
| 43 | ENSG00000224529 | AC093698.2 |  |  | 33897 | NaN | 0.4240 | No |
| 44 | ENSG00000277125 | AC211476.4 |  |  | 40490 | NaN | 0.3126 | No |
| 45 | ENSG00000236158 | RFC3P1 |  |  | 45281 | NaN | 0.2317 | No |
| 46 | ENSG00000237936 | AL353093.1 |  |  | 46045 | NaN | 0.2189 | No |
| 47 | ENSG00000256514 | AP003419.1 |  |  | 46088 | NaN | 0.2184 | No |
| 48 | ENSG00000213730 | POLD2P1 |  |  | 46754 | NaN | 0.2074 | No |
| 49 | ENSG00000255547 | RPA2P3 |  |  | 49116 | NaN | 0.1676 | No |
| 50 | ENSG00000232482 | AL357134.1 |  |  | 49526 | NaN | 0.1608 | No |
| 51 | ENSG00000249065 | PCNAP1 |  |  | 52056 | NaN | 0.1182 | No |
| 52 | ENSG00000218261 | AL591034.1 |  |  | 52536 | NaN | 0.1103 | No |
| 53 | ENSG00000254544 | PCNAP4 |  |  | 52840 | NaN | 0.1053 | No |
| 54 | ENSG00000250778 | AC004980.4 |  |  | 53838 | NaN | 0.0887 | No |
| 55 | ENSG00000214009 | PCNAP3 |  |  | 54078 | NaN | 0.0848 | No |
| 56 | ENSG00000226466 | RPA2P2 |  |  | 56081 | NaN | 0.0511 | No |
| 57 | ENSG00000228721 | AC016909.1 |  |  | 56445 | NaN | 0.0451 | No |
| 58 | ENSG00000179997 | AC010894.1 |  |  | 57169 | NaN | 0.0331 | No |
| 59 | ENSG00000230305 | AC004980.3 |  |  | 58644 | NaN | 0.0083 | No |
Table: GSEA details [plain text format]

  

Fig 2: MISMATCH\_REPAIR(HSA03430)      
 Blue-Pink O' Gram in the Space of the Analyzed GeneSet

  

Fig 3: MISMATCH\_REPAIR(HSA03430): Random ES distribution      
 Gene set null distribution of ES for **MISMATCH\_REPAIR(HSA03430)**
